# A High-Throughput Method for Screening for Genes Controlling Bacterial Conjugation of Antibiotic Resistance

**DOI:** 10.1128/mSystems.01226-20

**Published:** 2020-12-22

**Authors:** Hanna Alalam, Fabrice E. Graf, Martin Palm, Marie Abadikhah, Martin Zackrisson, Jonas Boström, Alfred Fransson, Chris Hadjineophytou, Linnéa Persson, Simon Stenberg, Matilda Mattsson, Payam Ghiaci, Per Sunnerhagen, Jonas Warringer, Anne Farewell

**Affiliations:** a Department of Chemistry and Molecular Biology, University of Gothenburg, Gothenburg, Sweden; b Centre for Antibiotic Resistance Research (CARe), University of Gothenburg, Gothenburg, Sweden; c Möller Data Workflow Systems, Gothenburg, Sweden; Marquette University

**Keywords:** high-throughput screening, antibiotic resistance, conjugation, horizontal transmission, plasmid, horizontal gene transfer, *Escherichia coli*

## Abstract

The rapid horizontal transmission of antibiotic resistance genes on conjugative plasmids between bacterial host cells is a major cause of the accelerating antibiotic resistance crisis. There are currently no experimental platforms for fast and cost-efficient screening of genetic effects on antibiotic resistance transmission by conjugation, which prevents understanding and targeting conjugation. We introduce a novel experimental framework to screen for conjugation-based horizontal transmission of antibiotic resistance between >60,000 pairs of cell populations in parallel. Plasmid-carrying donor strains are constructed in high-throughput. We then mix the resistance plasmid-carrying donors with recipients in a design where only transconjugants can reproduce, measure growth in dense intervals, and extract transmission times as the growth lag. As proof-of-principle, we exhaustively explore chromosomal genes controlling F-plasmid donation within Escherichia coli populations, by screening the Keio deletion collection in high replication. We recover all seven known chromosomal gene mutants affecting conjugation as donors and identify many novel mutants, all of which diminish antibiotic resistance transmission. We validate nine of the novel genes’ effects in liquid mating assays and complement one of the novel genes’ effect on conjugation (*rseA*). The new framework holds great potential for exhaustive disclosing of candidate targets for helper drugs that delay resistance development in patients and societies and improve the longevity of current and future antibiotics. Further, the platform can easily be adapted to explore interspecies conjugation, plasmid-borne factors, and experimental evolution and be used for rapid construction of strains.

**IMPORTANCE** The rapid transmission of antibiotic resistance genes on conjugative plasmids between bacterial host cells is a major cause of the accelerating antibiotic resistance crisis. There are currently no experimental platforms for fast and cost-efficient screening of genetic effects on antibiotic resistance transmission by conjugation, which prevents understanding and targeting conjugation. We introduce a novel experimental framework to screen for conjugation-based horizontal transmission of antibiotic resistance between >60,000 pairs of cell populations in parallel. As proof-of-principle, we exhaustively explore chromosomal genes controlling F-plasmid donation within E. coli populations. We recover all previously known and many novel chromosomal gene mutants that affect conjugation efficiency. The new framework holds great potential for rapid screening of compounds that decrease transmission. Further, the platform can easily be adapted to explore interspecies conjugation, plasmid-borne factors, and experimental evolution and be used for rapid construction of strains.

## Abstract (precis)

The rapid transmission of antibiotic resistance genes on conjugative plasmids between bacterial host cells is a major cause of the accelerating antibiotic resistance crisis. There are currently no experimental platforms for fast and cost-efficient screening of genetic effects on antibiotic resistance transmission by conjugation, which prevents understanding and targeting conjugation.

## INTRODUCTION

Antibiotic resistance, particularly in Gram-negative bacteria, is an accelerating crisis. In 2014, most areas of the world reported greater than 50% of Escherichia coli infections being resistant to third-generation cephalosporins, widespread resistance to fluoroquinolones, and accelerating resistance to third-generation carbapenems (1). Furthermore, in 2018 WHO identified carbapenem-resistant and third-generation cephalosporin-resistant *Enterobacteriaceae* as being a critical priority (2). Only a few new antibiotics against Gram-negative bacteria are in clinical trials, and the pipeline is insufficient to keep up with the rate of resistance emergence (3, 4). New approaches to this problem are therefore sorely needed. A major problem is that many antibiotic resistance genes can be transmitted horizontally into and between human pathogens (5). Horizontal transmission within pathogenic species, combined with selective pressure imposed by extensive antibiotic use, subsequently facilitates their extremely rapid spread and explosive antibiotic resistance evolution. The drastic decline in clinical potency of both frontline and “last-resort” antibiotics, including cephalosporins, carbapenems, and, most recently, colistins, is predominantly due to pathogen evolution by horizontal transmission of antibiotic defense factors (6, 7). Most often, horizontal transmission occurs via plasmid conjugation. The transferred conjugative elements can then be maintained as plasmids or integrated into the host chromosome (integrative conjugative elements).

Plasmids are self-replicating genetic modules capable of dissemination through conjugation and, to a lesser extent, transformation (5). More than 16,000 proteobacterial plasmids have been sequenced (8), and the associations of different conjugative plasmid families with various antibiotic resistances have been extensively explored in *Enterobacteriaceae* (9). Conjugation typically involves production of a pilus (encoded by the conjugative element) that attaches to a target cell and facilitates the transfer of the conjugative element to the recipient. It has recently been suggested that an effective approach to limit the spread of antibiotic resistance would be to inhibit conjugation of resistance-carrying plasmids (10, 11), by chemically blocking conjugation factors in either donors or recipients. However, plasmid-encoded conjugation factors are not well conserved across plasmids (7), decreasing their value as drug targets. Plasmid donation or receipt also depends on chromosomally encoded factors in donors and recipients that may be more promising as drug targets.

Few of these chromosomal genetic determinants of conjugation are known because of the absence of an approach that is sufficiently fast and cost-efficient for unbiased screening of tens of thousands of evolving bacterial populations. Measuring conjugation efficiency has traditionally relied on slow, meticulous mating assays that are prohibitively expensive and labor-intensive to scale up. Moderate-throughput designs were introduced to screen for conjugation effects in recipient cells but disclosed few/no genes of interest (12, 13). Here, we develop, implement, and validate a high-throughput experimental evolution framework to monitor the conjugation of resistance-carrying plasmid donor libraries to a recipient cell population in near-real time. The framework can accommodate screening of a wide variety of clinically relevant plasmids, species, and environments, and we expect it to become invaluable in the search for chemical inhibitors of conjugative spread of antibiotic resistance.

## RESULTS

### A high-throughput platform for measuring conjugation of antibiotic resistance plasmids.

**FIG 1:**
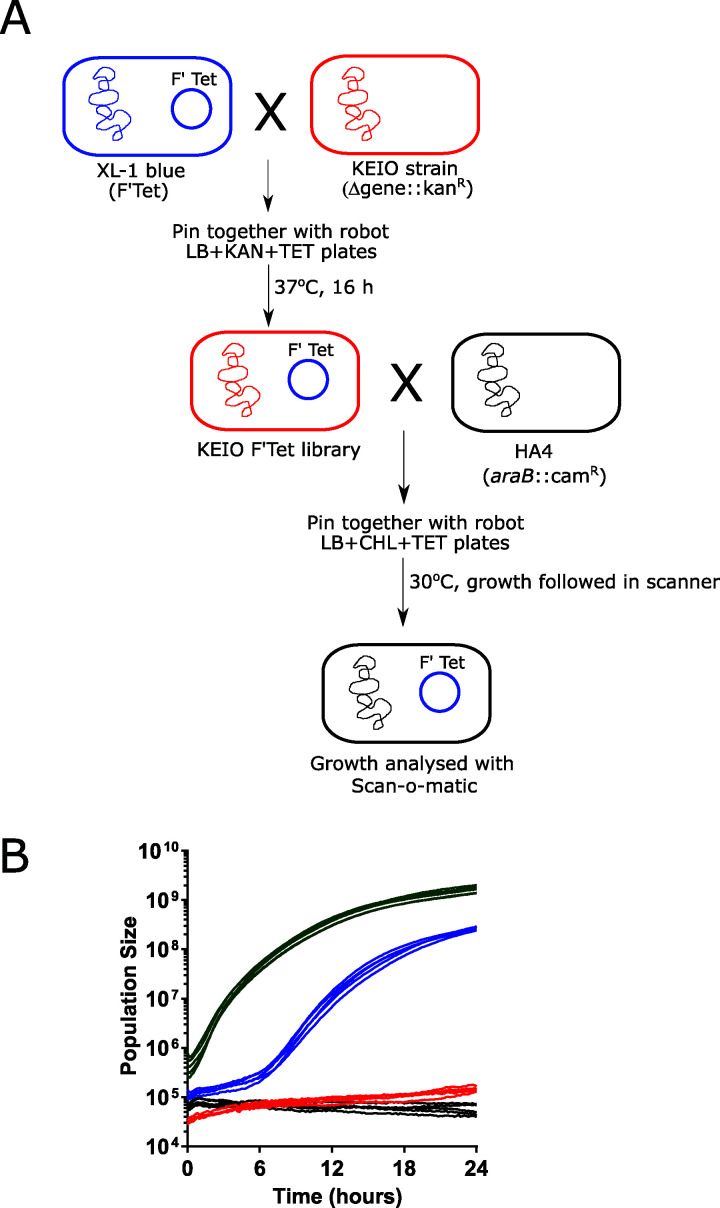
Experimental scheme for screening strains for conjugative efficiency. (A) Construction and screening of donor library. The donor library constructed by mating XL1-Blue (F′ Tet^r^) with the Keio collection carrying a kanamycin resistance gene in place of almost 4,000 genes is shown at the top. Resulting donor (F′ Tet^r^) and recipient (chromosomal Cam^r^) strains were then grown separately on appropriate preculture plates and then pinned robotically in a 1536 format to a selective plate (Tet Chl) that allows only transconjugants to grow. Plates were placed in a flatbed scanner and scanned every 10 min for 24 h at 30°C. Data were then analyzed with Scan-o-Matic as described in the text. (B) Growth of transconjugants formed on selective plates. Blue denotes growth of spots pinned with HA4 (chromosomal Cam^r^) and HA14 (F′ Tet^r^) together, showing growth of the resulting transconjugants which occurs after a lag compared to HA5 (Cam^r^ Tet^r^) (green), which grows with no detectable lag. Negative controls HA4 alone (red) and HA14 alone (black) are shown. Five representative graphs are shown for each taken from two technical replicates of 768 biological replicates (HA14 × HA4), 384 biological replicates (HA5), and 192 biological replicates (negative controls).

We have designed a platform capable of accurately measuring the conjugative transmission of plasmid-borne antibiotic resistance factors at high throughput. We robotically construct E. coli donor strains, collect donor and recipient cells from distinct source plates, and deposit them as a mixed population on a target plate that is doubly selective for two noninterfering, bacteriostatic antibiotic resistances (Fig. 1A). Recipient cells carry a nontransmissible chromosomally encoded antibiotic resistance, while donors host a conjugative plasmid with the resistance to be transferred. Therefore, only recipient cells that have received a plasmid from a donor, i.e., transconjugants, will divide on the doubly selective plate. The growth lag of the mixed population will reflect the time to conjugate the plasmid and express its resistance gene. Fixing the recipient genotype, the time required to express the resistance gene becomes a constant. Conjugation time variation therefore equals lag time variation. To measure lag time, we adopted a recently introduced platform, Scan-o-Matic (14), originally developed for surveying Saccharomyces cerevisiae colony population size expansion in high throughput. We deposited 1,152 mixed populations on each plate, maintained plates on flatbed scanners in thermostatic cabinets, and acquired transmissive light images every 10 min. Colonies were identified, background was subtracted, and pixel intensities were extracted and finally transformed into population cell counts in a fully automated procedure. To establish baseline parameters, we mated E. coli donor cells carrying an F-plasmid with tetracycline resistance to E. coli recipients with chloramphenicol resistance in a conjugation neutral locus (Δ*araB*::Cam^r^) in 768 mixed populations (Fig. 1B). We obtained an average lag time of approximately 5.46 h (at 30°C), with only small spatial variation across the plate (coefficient of variation = 9%). Pure donor and pure recipient cell populations uniformly failed to grow, and an E. coli strain with both resistance markers grew with no detectable lag phase (Fig. 1B).

### Comprehensive view of donor functions controlling F-plasmid conjugation.

**FIG 2:**
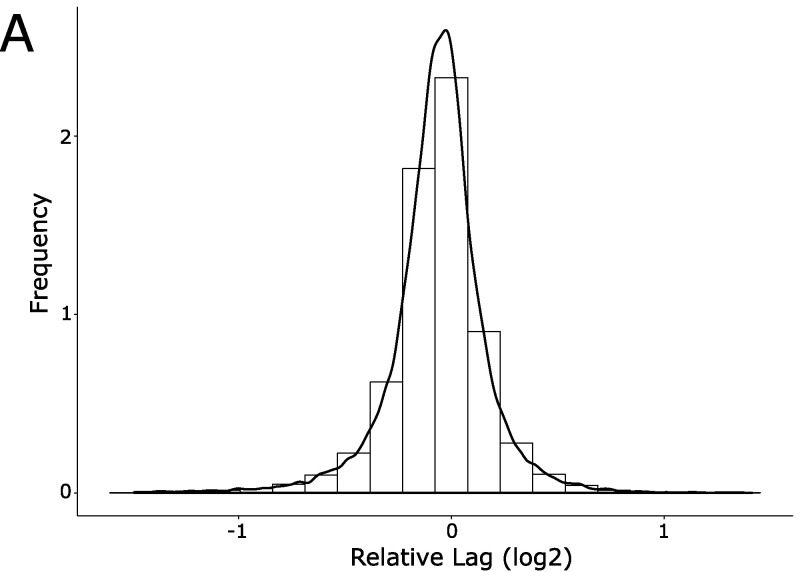

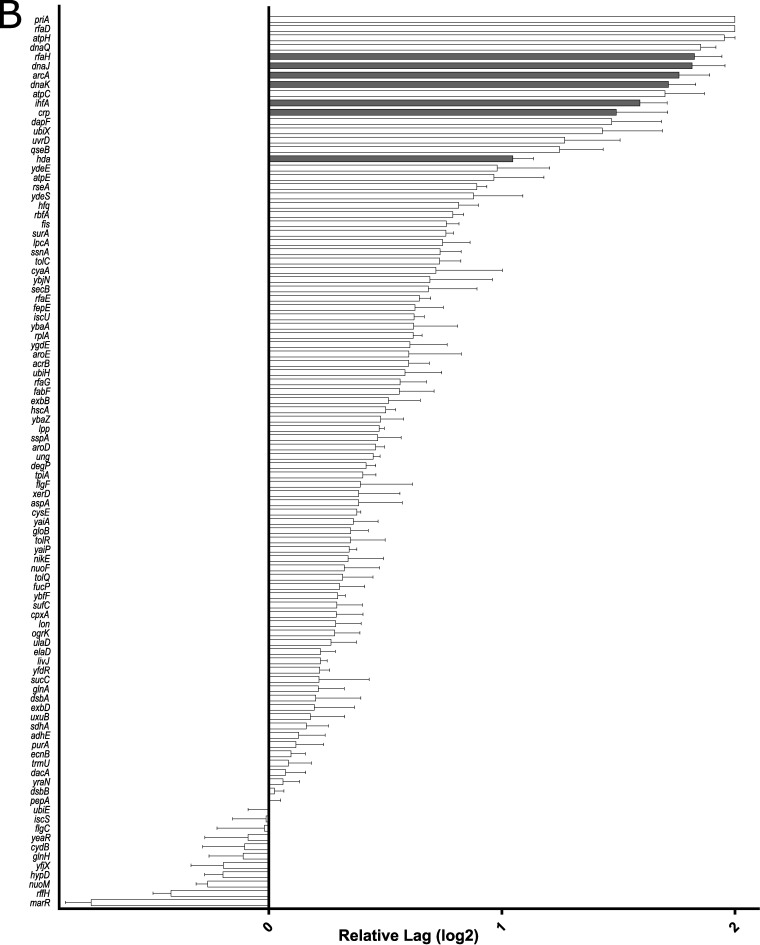
Conjugative efficiency of the Keio collection. (A) Growth lag time of the entire Keio collection in the conjugation assay. The growth lag of each curve is expressed relative to the nearby control and the log_2_ value calculated. Shown is the frequency plot for the collection: a positive value indicates that the strain has a longer lag period and a negative number indicates it is shorter than the control mating. Data are derived from four biological replicates done with two technical replicates as described in the text. (B) Growth lag time of the top candidates. Conjugation efficiency screening was repeated with 96 strains at higher replication (18 replicates). Strains were chosen as described in the text. Plotted is the mean of the values with the standard error of the mean. Especially in the top candidates, many of the replicates had no detectable conjugation. For comparison, we have set values in this data set with no measurable growth lag to a value of 2, which corresponds to a mating lag time of four times the local control. Two strains had no measurable conjugation in any of the replicates (*priA* and *rfaD*). Two of the strains (*aroD* and *crp*) had fewer replicates (12). Previously known conjugation-deficient mutants are indicated with gray bars.

**FIG 3:**
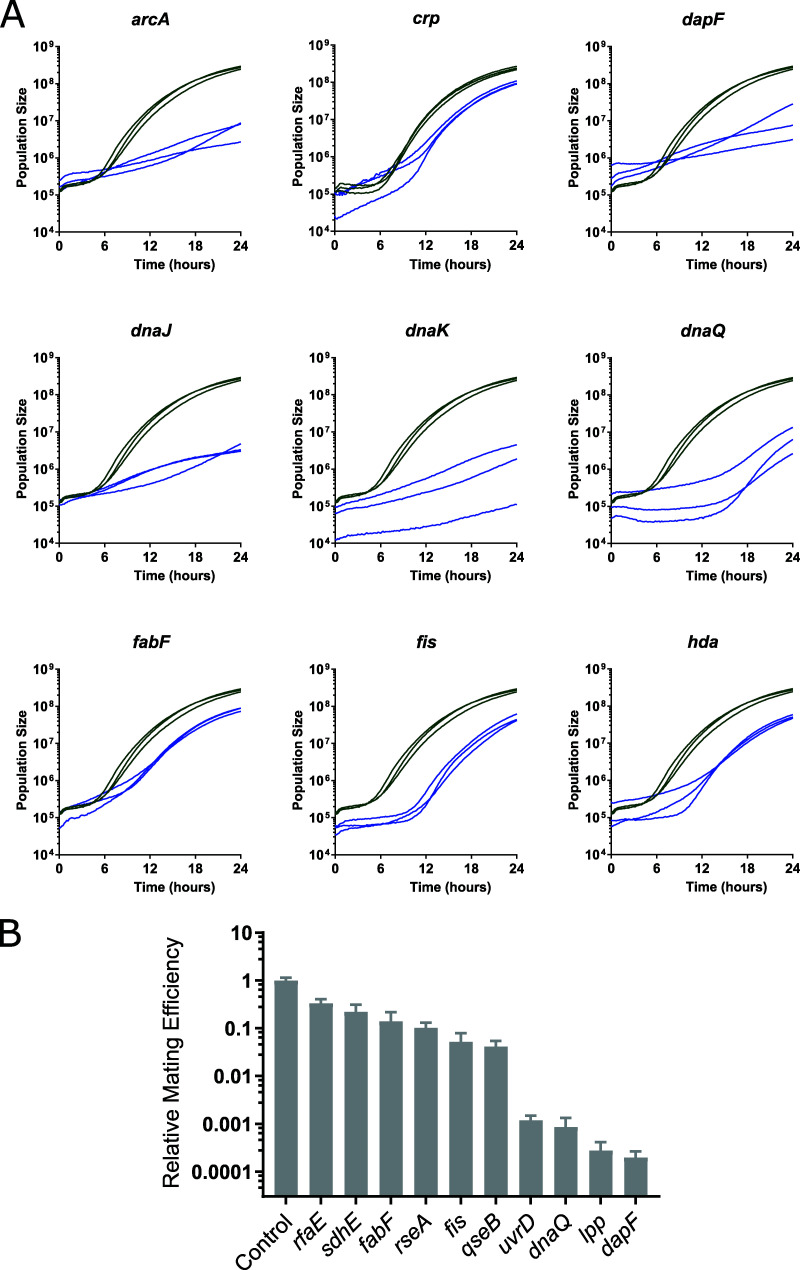
Conjugative efficiency from strains with detectable defects in mating. (A) Representative growth curves from 5 previously known (*arcA*, *crp*, *dnaJ*, *dnaK*, and *hda*) and four newly identified (*dapF*, *dnaQ*, *fis*, and *fabF*) conjugation-deficient mutants. The deleted gene in each strain is indicated. Three curves were taken from the plate screening experiments for each mutant as indicated (blue) and three nearby control mating results (green). The curves are representative of the 6 biological replicates each done with three technical replicates (*n* = 18). (B) Liquid mating assay results from 9 newly identified conjugation-deficient strains. The gene deleted in each mutant is indicated, and the average mating efficiency (number of transconjugants per number of donor cells divided by the corresponding value of a control mating done on the same day) in a 30-min liquid mating assay with 4 to 6 biological replicates (each with two technical replicates) over 2 to 3 different days is shown. Error bars indicate the standard error of the mean. *P* values were calculated using a one-sided Student *t* test: all results had a *P* value of <0.001.

Next, we introduced the tetracycline resistance-carrying F-plasmid into the 3,908 deletion strains of the E. coli Keio library (15) by mating to a fixed XL1-Blue genotype carrying F′ Tet^r^ followed by multiple rounds of double selection (Fig. 1A). We subsequently mated the Keio donor library to a fixed recipient genotype (HA4; chloramphenicol resistance) at moderate replication (*n *= 8; on two plates), while monitoring the conjugation using the high-throughput platform (14). We deposited 1,152 populations on each plate, interleaving 384 genetically identical controls (HA14 × HA4) in every fourth position to control for any systematic spatial effects. We extracted the lag time for each experimental population, normalized it to that of neighboring controls, and expressed the ratio on a log_2_ scale. Positive numbers reflect longer lag time and delayed conjugation compared to the control. Overall, donor gene effects on conjugation were symmetrically distributed (μ = −0.062, σ = 0.23) around the control mating mean, with extremes being more common than expected from a normal distribution and somewhat more likely to correspond to delayed conjugation (Fig. 2A; see also Fig. S1 in the supplemental material). We selected 58 of the most affected gene deletions for further validation as the most promising drug targets, as well as 28 weaker hits down to rank 236 to test reproducibility also for more marginal effects. We retested these 86 candidates in a high-replication (*n* = 18) secondary screen (Fig. 2B and Table S1) along with 6 very low-ranked candidates as negative controls. Gene effects on conjugation generally agreed well (*R*^2^ = 0.56) between the primary and secondary screen with 71 of the 86 strains chosen from the first screen giving statistically significantly longer lag times than the control mating (Fig. 2B and Table S1; example graphs are shown in Fig. 3A and Fig. S2). Two further mutants (*aroD* and *crp*) were identified as deficient in conjugation from a screen of 108 mutants that were done after the primary screen. Importantly, we recovered all previously described chromosomal mutants known to affect F-plasmid (or F-like plasmid) conjugation: *arcA* (16), *crp* (17), *hda* (18), *dnaK* (19), *dnaJ* (19), *ihfA* (20), and *rfaH* (21) (indicated by gray bars in Fig. 2B). We also identified >50 novel genes whose deletion strains were consistently defective in F-plasmid donation in both screens (Table S1). The mutants’ encoded gene products covered a range of cellular functions but were disproportionately likely to mediate DNA replication (6 proteins, *P* < 10^−3^), chaperone or protein folding functions (6 proteins, *P* < 10^−4^), and lipopolysaccharide core biosynthesis (4 proteins, *P* < 0.001) (Fisher’s exact test, EcoCyc [22]; Table S1). We found very few strains that appeared to have increased conjugation efficiency, but this is likely a technical issue; it is difficult to measure shorter lags in the current experimental setup. We did rescreen the 6 fastest strains (*marR*, *rffH*, *yfjX*, *nuoM*, *glnH*, and *yeaR*) observed in the primary screen, and all were significantly faster than the control (*P* < 0.05). These were not further examined.

10.1128/mSystems.01226-20.2 FIG S1 Quantile-quantile plot Q showing the empirically observed quantiles of gene effects on conjugation (*y* axis) as a function of quantiles expected from a normal distribution with the same mean and variance as the empirical distribution (*x* axis). Note that extreme gene effects are more common than expected from a normal distribution (red line). Download FIG S1, PDF file, 0.2 MB. Copyright © 2020 Alalam et al. 2020 Alalam et al. This content is distributed under the terms of the Creative Commons Attribution 4.0 International license.

10.1128/mSystems.01226-20.3 FIG S2 Representative transconjugant growth curves from matings with 12 conjugation-deficient mutants. The deleted gene in each strain is indicated. Three curves were taken from the plate screening experiments for each mutant as indicated (blue) and three nearby control mating results (green). Download FIG S2, PDF file, 0.1 MB. Copyright © 2020 Alalam et al. 2020 Alalam et al. This content is distributed under the terms of the Creative Commons Attribution 4.0 International license.

10.1128/mSystems.01226-20.8 TABLE S1 Relative conjugation efficiency of selected mutant donors. Shown is the relative lag times (log_2_ scale) of the indicated Keio mutant donor in a high-throughput conjugation assay. Data were calculated as described in the text and Fig. 2. (a) Mutants are ranked according to the median effect on lag phase in the initial screen. (b) Gene product was retrieved from ecocyc.org (I. M. Keseler, A. Mackie, A. Santos-Zavaleta, R. Billington, et al., Nucleic Acids Res 45:D543–D550, 2017, https://doi.org/10.1093/nar/gkw1003). (c) For comparison, mutants which showed no conjugation in a given screen were assigned a value of 2.0, indicating that the lag phase was at least longer than 4 times the relevant control. (d) Significance is reported as the false-discovery rate (FDR)-adjusted *P* value from a one-sample Wilcoxon test performed versus a value of zero. *, these strains had 50% or more of the results in the first screen exhibiting no conjugation. Download Table S1, DOCX file, 0.04 MB. Copyright © 2020 Alalam et al. 2020 Alalam et al. This content is distributed under the terms of the Creative Commons Attribution 4.0 International license.

We considered several sources of confounding effects. First, poor growth of the donor strains could appear to give a conjugation-deficient phenotype. *rimM* and *rnt* strains were initially scored as conjugation deficient but were discarded as likely false positives due to their very poor growth on the background LB medium (they do not form detectable single colonies on LB medium in 24 h). To further test the effect of growth rate, we measured the growth rates of 81 donor strains used in the secondary screen and showed that there was no correlation between growth rate (on media selective for the plasmid) and the lag time during mating (*R*^2^ = 1e^−5^, Fig. S3), suggesting that poor growth was not the main cause of poor mating efficiency. Second, we considered that some strains could be hypersensitive to the chloramphenicol included in the mating plates as counterselection. We cross-referenced our candidates with the 19 strongly chloramphenicol-hypersensitive deletions previously identified (23): in our secondary screen, we recovered only 5 of these mutants (*tolC*, *flgF*, *rfaG*, *rfaE* and *acrB*). Thus, these five would need to be independently verified in the absence of the antibiotic (*rfaE* was verified [see below]). Also, note that the *marR* mutant, which was identified as having a shorter-than-normal lag time, is known to be resistant to chloramphenicol (24), which could explain why it appeared to have a short lag time. Third, we considered that some deletions could have effects on downstream or overlapping genes. This is difficult to resolve completely with incomplete genomic information of promoter structure, but we removed one top candidate, *yjjY*, because it is a deletion of a small open reading frame (ORF) that overlaps the *arcA* promoter (25), which is known to affect conjugation (16). A last source of confounding data, secondary mutations in the collection, is addressed below.

10.1128/mSystems.01226-20.4 FIG S3 Growth rates versus lag time. The growth rate of 81 Keio strains carrying the F′ that were identified in the initial screen as conjugation defective is plotted against the lag time observed in the secondary screen. Both are plotted as the log_2_ value relative to the control strain or control mating. Growth rate was measured independently on plates containing only kanamycin and tetracycline to select for the plasmid in two experiments each with 12 biological replicates. The dashed red lines indicate the “0” value where there is no difference between the growth rate or lag time of the mutant and that of the control strain or control mating. The blue line shows the predicted correlation (*R*^2^ = 1e^−5^). Raw data are available in Table S1 (lag time) and at https://github.com/annefarewell/Conjugation-factors-F-plasmid (growth rates). Download FIG S3, PDF file, 0.1 MB. Copyright © 2020 Alalam et al. 2020 Alalam et al. This content is distributed under the terms of the Creative Commons Attribution 4.0 International license.

To exclude confounding cross-contamination and strain construction errors, we validated the absence of the expected gene in 36 of the conjugation-deficient deletion mutants by PCR (Table S2). All but one were confirmed to be deleted (our copy of the *sdhE* mutant could not be verified and has been removed from our data set). We next validated the conjugation defects of nine novel mutants in an independent, liquid mating assay. These nine were chosen to span the degree of defects observed in our screen, from strong effects to weaker effects. Unlike the plate assays, conjugation in liquid mating assays occurred in the absence of antibiotics. Because the distinction between liquid and solid medium conjugation is important, with liquid matings often detecting additional effects (i.e., mating pair stabilization [26]), we did not expect a direct quantitative correlation (e.g., compare mating deficiency in the *lpp* mutant [Fig. 3B versus Fig. S2]). Nevertheless, all nine mutants showed conjugation deficiencies also in liquid, ranging from 0.02% to 26% of the conjugation efficiency of the wild type (Fig. 3B). Among the 9 mutants, we also saw significant effects even in the mutant with the weakest effect on conjugation tested (*rfaE*) (*P* < 0.001, Fig. 3B); this indicates that mutants ranked down to position 74 in our initial screen are promising candidates for conjugation defects and should not be disregarded. Further, this experiment shows that the presence of antibiotics during the original screen did not create significant artifacts (e.g., altered gene expression or poor growth).

10.1128/mSystems.01226-20.9 TABLE S2 Primers used to check Keio strains. Download Table S2, DOCX file, 0.02 MB. Copyright © 2020 Alalam et al. 2020 Alalam et al. This content is distributed under the terms of the Creative Commons Attribution 4.0 International license.

We also tested the ability of 12 of our strains with defective conjugation to form plaques when bacteriophage Qβ was spotted on a lawn of the strain. Because Qβ uses the F-pilus as a receptor (27, 28), we reasoned that an absence of plaques would suggest that an E. coli deletion mutant lacks F-pili, explaining its conjugation efficiency in a direct way. We found that four of the tested deletion mutants, those missing *arcA*, *dapF*, *qseB*, and *dnaQ*, respectively, could not form plaques (Table S3), indicating that their conjugation deficiency may be due to an inability to form, or anchor, intact F-pili. Because the assay is qualitative rather than quantitative, some of the other mutants forming plaques could also have defects in or decreased numbers of F-pili.

10.1128/mSystems.01226-20.10 TABLE S3 Efficiency of plating (EOP) of phage Qβ on Keio mutant strains carrying F′. Tenfold dilutions of phage Qβ (∼10^11^-PFU/ml stock concentration) were spotted on a soft agar lawn of the indicated strains. +++ indicates EOP equivalent to the wild-type positive control (100%), 0 indicates no plaques obtained (<10^−6^%). There was no significant difference between the strains labeled with an EOP of +++. Download Table S3, DOCX file, 0.02 MB. Copyright © 2020 Alalam et al. 2020 Alalam et al. This content is distributed under the terms of the Creative Commons Attribution 4.0 International license.

While we stringently validated the defect in conjugation in nine deletion strains, we could not exclude the possibility that *trans* effects on conjugation from secondary mutations at other genomic loci or *cis* effects on adjacent loci from the deletion cassette insertion could confound the assignment of causality. We therefore first used transduction and then a complementation assay to confirm that the missing gene is required for efficient conjugation. We transduced five of the nine gene deletions with validated conjugation defects to BW25113 (wild type). We found that the conjugation defects of the *fis* and *rseA* gene deletions were transduced together with the mutation and that their effects on conjugation are therefore likely caused by the gene deletion (Fig. S4). However, although we could transduce Δ*dapF*::Kan and Δ*uvrD*::Kan and show that other phenotypes (lysine auxotrophy and UV sensitivity, respectively) known to be caused by the gene deletions were cotransduced together with the mutations, the transduced strains were not defective in conjugation. We were unable to transduce the *dnaQ* mutation due to its apparent P1 resistance.

10.1128/mSystems.01226-20.5 FIG S4 Mating assays of the transduced Keio mutants compared to the original strains. Shown is the average growth curve of transconjugants from four mating experiments (the number of biological replicates in each experiment is indicated below). Blue, control donor (HA14, 4 biological replicates); green, original Keio strain (10 biological replicates); red, transduced Keio mutation into BW25113 (8 biological replicates). Download FIG S4, PDF file, 0.03 MB. Copyright © 2020 Alalam et al. 2020 Alalam et al. This content is distributed under the terms of the Creative Commons Attribution 4.0 International license.

We also tried to complement five gene deletions by cloning in each gene under the control of its native promoter on a plasmid and introducing each plasmid into its corresponding gene deletion strain (Text S1 and Fig. S5). We successfully complemented the *arcA* conjugation deficiency by reintroducing *arcA*, confirming our and previous findings that *arcA* is required for F-plasmid conjugation. We also successfully complemented the *rseA* mutant. RseA is an anti-sigma factor that represses activation of the Sigma-E (σ^E^) pathway and is the major regulator of this pathway (29, 30). Three of the mutants could not be complemented for the conjugation defect with the plasmids we constructed: *dapF*, *uvrD*, and *dnaQ*. Lysine auxotrophy and UV sensitivity were complemented in *dapF* and *uvrD* mutants, respectively. Although we cannot exclude that the expression of these genes from a plasmid is not exactly equivalent to expression from their chromosomal loci, it is also quite possible that these gene deletion strains in the Keio collection contain secondary mutations that account for the conjugation defects, as discussed below.

10.1128/mSystems.01226-20.1 TEXT S1 Supplemental materials and methods. Download Text S1, DOCX file, 0.02 MB. Copyright © 2020 Alalam et al. 2020 Alalam et al. This content is distributed under the terms of the Creative Commons Attribution 4.0 International license.

10.1128/mSystems.01226-20.6 FIG S5 Average transconjugant growth curves of complemented strains. Shown is the average growth curve from four experimental replicates with 32 biological replicates of each of the indicated mutants carrying a vector plasmid (blue) compared to the mutant carrying the appropriate complementation plasmid (red) and a control mating with HA14 (green). Download FIG S5, PDF file, 0.05 MB. Copyright © 2020 Alalam et al. 2020 Alalam et al. This content is distributed under the terms of the Creative Commons Attribution 4.0 International license.

## DISCUSSION

We designed a high-throughput platform for measuring conjugation of antibiotic resistance plasmids. We demonstrated its utility by identifying all previously known E. coli genes that control F-plasmid donation, as well as many novel conjugation genes not previously linked to antibiotic resistance transmission. The novel conjugation-deficient mutants span an unexpectedly wide range of functions. Some of these can be rationally explained, e.g., those altering the cell surface, e.g., lipopolysaccharide. Lipopolysaccharide mutants have previously been reported to have defects in acting also as conjugation recipients (12) and may affect the mating pair interaction. Other mutants have no obvious connection to conjugation and likely act indirectly, e.g., by controlling expression (transcription, translation), folding (chaperone), and energy supply for conjugation components. The deletion mutants identified here will also need to be confirmed to exclude the possibility of secondary mutations.

IncF plasmids are narrow-host-range (limited to *Enterobacteriaceae*) plasmids but are highly diverse within their group and associated with extended-spectrum beta-lactamase (ESBL)-producing E. coli; an IncFII plasmid containing CTX-M15 ESBL (extended-spectrum beta-lactamase gene) was likely a contributor to the emergence and establishment of the globally dominant E. coli sequence type 131 (ST131) (31). Nevertheless, IncF plasmids are not the only plasmids of high clinical concern, with Inc A/C, L/M, N, I1, and HI2 plasmids all representing major challenges (7). Whether the same, or distinct chromosomal, factors control transmission of non-IncF plasmids is unknown but critical to any drug development effort. We note that no conceptual challenges prevent identifying the determinants of Inc A/C, L/M, N, I1, and HI2 plasmid transmission, using the introduced platform.

The methodology we have developed is highly adaptable for similar experimental designs targeting conjugation in other bacteria, or cross-species conjugation. Strong coloration in bacteria or background medium or massive secretion of polysaccharides can interfere with correct population size estimations since the detection depends on visible light and will, to some degree, affect conjugation time estimates; we have encountered no other method-related factors that constrain the general applicability of the platform. Further, the platform is ideal for long-term studies of bacterial evolution because of the accuracy of population size measurements, large number of replicates, and ease of use. We note the fact that some of the gene deletions associated with conjugation defects could not be confirmed as causative when transducing the gene deletion to a wild-type background or when complementing the deleted gene by reintroducing it on a plasmid. While this does not cast a shadow on the method as such, it calls for caution when applying it to the Keio gene deletion collection, and perhaps in a broader context when using such collections. Deletion strain construction is a mutagenic process *per se*, and the many cell divisions associated with storing and propagating such collections add opportunities for confounding background mutations to emerge. The consequences of this are well documented in yeast, where confounding effects from both point and structural mutations at secondary sites are common in deletion collections (32–35), but have not been extensively explored in E. coli.

Here, we could not transduce or complement the conjugation defects of *dapF*, *uvrD*, and *dnaQ*, despite stringently confirming the conjugation defects of their corresponding Keio collection deletion strains in multiple validation assays. For two of these mutants, the occurrence of secondary mutations indeed seems likely. *dnaQ* mutants are known to require secondary mutations in *dnaE* to be viable in Salmonella enterica serovar Typhimurium (36), and we had difficulties transducing this mutation to a fresh wild-type background. Indeed, when we sequenced *dnaE* in the original Keio *dnaQ* deletion strain, it carries a single amino acid change (Q429P), and it is possible that mutations at other loci also exist. Further work will need to be done to determine whether this, or other mutations, acts as a suppressor and causes the conjugation defect. Furthermore, *dnaQ* mutants, which lack DNA polymerase III proofreading function, have intrinsically high mutation rates that often lead to secondary mutation accumulation and error catastrophes with detrimental effects on fitness (37).

The essentiality of *dapF* is controversial. We have noted that though a *dapF* knockout is viable in BW25113, it cannot be transduced to other genetic backgrounds directly, showing that other mutations are required for viability. It is quite possible that such mutations, alone or in combination with the *dapF* deletion, account for the conjugation-deficient phenotype. However, we were able to show that the lysine auxotrophy of the *dapF* mutant was complemented in our strain, suggesting that any secondary mutation acts independently to give the conjugation-deficient phenotype. Finally, *uvrD* could be complemented for UV sensitivity but not conjugation efficiency. We do not have suggestions that this strain would frequently gain secondary mutations from the literature.

The confounding effects from secondary mutations in the Keio collection are not a limitation of our method and do not detract from its value. However, the fact that these effects are not rare means that we cannot take the results from the current screen at face value and that follow-up work is required to translate the lists of both candidate and validated hits into a true biological understanding of conjugation. And, in a broader perspective, they serve as a cautionary tale that phenotypes obtained using the Keio collection should be carefully validated. Indeed, this is not an issue confined to the Keio collection. Numerous studies/databases have aimed to define the set of essential genes in E. coli (reviewed in the work of Martinez-Carranza et al. [38]). These studies vary dramatically in the number of essential genes identified, ranging from 302 to 620 with only 164 genes found in all the studies. Even excluding the one study that found many more essential genes (365 unique to that study [39]), there is only approximately a 50% overlap in the essential genes identified from the remaining studies. Part of the reason for this disparity is the various methodologies used to make the mutant collections, but the selection of secondary mutations or variants at other sites likely accounts for much of this variation.

The fact that *rseA* mutants exhibit poor conjugation suggests that activation of the Sigma-E pathway, or a downstream target of this pathway, is inhibitory to conjugation. Extracytoplasmic (envelope) stress is sensed by several regulatory pathways in E. coli, most notably by the CpxAR two-component regulon and the Sigma-E regulon (40). There are several reported interactions between these pathways and the process of conjugation. Expression of the conjugative machinery is thought to cause extracytoplasmic stress (41), and the F-plasmid-encoded protein TraR has been shown to facilitate transcription of Sigma-E promoters (42), while F-like plasmids have been shown to induce the CpxAR regulon (43). This suggests that F-encoded functions have evolved to minimize extracytoplasmic damage caused by conjugation. In addition, it has been shown that the CpxAR regulon represses expression from the major F-plasmid P_Y_ promoter via TraJ (44, 45); thus, the cell minimizes the additional stress of conjugation during extracytoplasmic stress conditions. We suggest that similarly to CpxAR, it is likely that the deletion of RseA results in decreased conjugation (presumably through its effect on Sigma-E) to spare the cell during extracytoplasmic stress.

In summary, we report a high-throughput method for measuring conjugation efficiency in E. coli. The method can be used in the future to measure the effects of environmental factors on conjugation as well as screening of mutant collections. This added tool will aid research into conjugation with an aim to minimize the spread of antibiotic resistance.

## MATERIALS AND METHODS

### Strains and media.

Strains are listed in Table 1. Chemicals were purchased from Sigma-Aldrich, Inc., unless otherwise noted. LB medium was routinely used (5 g/liter yeast extract, 10 g/liter tryptone, and 10 g/liter NaCl; 15 g/liter agar added as needed). When appropriate, the medium was supplemented with chloramphenicol (30 μg/ml), kanamycin (50 μg/ml), and/or tetracycline (10 μg/ml), here referred to as CHL, KAN, and TET, respectively. M9 minimal medium (46) was used for testing auxotrophy of the *dapF* mutant with or without the addition of 0.25 mM lysine. Liquid cultures were grown in a rotary shaker at 37°C at 220 rpm. Strain HA4 was constructed by replacing *araB* in MG1655 (47) with a chloramphenicol resistance marker using primer FWD araB CHL (ATTGGCCTCGATTTTGGCAGTGATTCTGTGCGAGCTTTGGCGGTGGACGTGTAGGCTGGAGCTGCTTC) and primer RVS araB CHL (AAGTTGGAAGATAGTGTTGTTCGGCGCTCATCGCCCATTGCTGATAGCGATGGGAATTAGCCATGGTCC) to amplify the Cam^r^ gene from pKD3, followed by transformation into MG1655/pKD46 carrying Lambda Red and P1 transduction into BW25113 as previously described (48). Strain HA5 was constructed by conjugating XL1-Blue (Stratagene, Inc) with HA4 on solid LB for approximately 3 h and then selecting for transconjugants on LB TET CHL. Strain HA14 was retrieved from the donor library (see below) and streaked on LB TET KAN.

**TABLE 1:** E. coli strains used in this work

Strain	Relevant genotype	Resistance	Source or reference
BW25113	[Δ(*araD-araB*)*567* Δ(*rhaD-rhaB*)*568* Δ*lacZ4787* (::rrnB-3) *hsdR514 rph-1*]	None	15
HA4	BW25113 *araA*^+^ *araC*^+^ Δ*araB*::Cam^r^	Chromosomal Cam^r^	This work
HA5	BW25113 *araA*^+^ *araC*^+^ Δ*araB*::Cam^r^ [F′ *proAB lacI*^q^*Z*ΔM15 Tn*10* (Tet^r^)]	Chromosomal Cam^r^; plasmid Tet^r^	This work
HA14	BW25113 Δ*argC*::*kan* [F′ *proAB lacI*^q^ *ZΔ*M15 Tn*10*]	Chromosomal Kan^r^; plasmid Tet^r^	This work
XL1-Blue	*recA1 endA1 gyrA96 thi-1 hsdR17 supE44 relA1 lac* [F′ *proAB lacI*^q^*ZΔ*M15 Tn*10*]	Plasmid Tet^r^	Stratagene

### Initial testing of the system.

Frozen 96-well stock plates of HA4 (Cam^r^ recipient), HA14 (control donor F′ Tet^r^), and HA5 (control Cam^r^ Tet^r^ strain) were created by mixing an overnight culture with glycerol to a final concentration of 15% and adding 175 μl to each well. Precultures were prepared by pinning from these 96-well plates to positions as described in Fig. S6A in the supplemental material. Precultures were incubated at 30°C for 16 h. Subsequently, the control HA5 preculture was transferred to an LB TET CHL plate (prewarmed to room temperature) using a 1,536-pin short pad and an HDA RoToR robot (Singer Ltd, United Kingdom) followed by the recipient preculture and then the donor preculture to the same LB TET CHL plate, resulting in positions containing matings of HA4 × HA14, negative controls, and control growth positions (HA5) (see Fig. S6A). The pinned plate was then moved to an Epson Perfection V800 photo scanner (Epson Corporation, United Kingdom) in a temperature- and humidity-controlled cabinet, and a consecutive series of images was produced at a periodicity of 10 min at 30°C over 24 h. The lag of each growth curve was calculated as described below.

10.1128/mSystems.01226-20.7 FIG S6 (A) Robot pinning coordinates and pinning scheme for test of conjugation system. Pinning coordinates are identified by a letter and a number: each coordinate represents a 96-well plate, and a total of 16 96-well plates can fit per plate (1,536 spots in total). “Mating” indicates positions where HA4 was pinned together with HA14, negative controls are indicated by “Donor” or “Recipient,” and “HA5 control” indicates the position of the positive growth control (Tet^r^ Cam^r^). To create this pattern, three preculture plates were pinned together. The recipient preculture of HA4 was pinned from a 96-well plate into positions A1 to A4, C1 to C4, and B1 + D3 of an LB CHL plate. Donor precultures were prepared by pinning from an HA14 96-well plate to positions A1 to A4, C1 to C4, and B3+D1 of an LB TET KAN plate. Positive-control precultures were prepared by pinning from an HA5 96-well plate to every fourth position (B2, B4, D2, and D4) of an LB TET CHL plate. A 1,536-pin pad was used to pin from the three precultures onto an LB TET CHL plate. The resulting format is shown, and the data from this experiment are in Fig. 1B. (B) Donor library construction. Robot pinning coordinates and pinning scheme for donor library construction. The pinning scheme for donor library construction is indicated. The pinning coordinate for the mating at A1 will contain the transconjugants, while the coordinates for the recipient’s negative control (the Keio mutant) and the donor’s negative control are indicated at positions C2 and C3, respectively. The remaining positions are not used. (C) Donor library screening for conjugation efficiency. The format of the Keio screen matings and controls is shown. Two different sets of 96 Keio mutants were pinned onto one plate as indicated by K1 and K2. “Mating” indicates positions where a Keio donor strain was pinned together with the recipient strain (HA4), control matings of HA14 and HA4 are included in every fourth position, and negative controls for the Keio (donor K1 and K2), HA14 (control donor), and HA4 (recipient) strains are indicated. This plate was created by first pinning strains onto two preculture plates and then using a 1,536-pin pad to pin the final pattern onto an LB TET CHL plate. The precultures were done as follows. Donor preculture: positions A1 to A4 and B1 are pinned with the first donor Keio plate (P1), positions C1 to C4 and D1 are pinned with the second donor Keio plate (P2), and every fourth position and B3 are pinned with HA14 on an LB TET KAN plate. On the recipient preculture LB CHL plate, all positions except B1, B3, and D1 are pinned with plate HA4. Results from this experiment are shown in Fig. 2. Download FIG S6, PDF file, 0.1 MB. Copyright © 2020 Alalam et al. 2020 Alalam et al. This content is distributed under the terms of the Creative Commons Attribution 4.0 International license.

### Construction of the donor Keio library.

The Keio donor library was constructed by conjugating the F-plasmid from XL1-Blue to the Keio mutants (15). An HDA RoToR robot (Singer Ltd, United Kingdom) was utilized to construct the donor library by pinning cells from 96-well plates onto solid LB TET KAN plates using 96-long pads (Singer Ltd, United Kingdom). A 96-well plate of XL1-Blue was created by mixing an overnight culture of XL1-Blue grown in LB TET with glycerol (20% final concentration) and pipetting 175 μl into a 96-well plate. The Keio 96-well plate was created similarly. Each Keio plate was pinned twice (positions A1 and C2, in each tetrad of positions); then the XL1-Blue 96-well plate was also pinned twice (positions A1 and C3) followed by incubation at 37°C overnight. This created a mating position at A1 and negative controls for each strain (Fig. S6B). Only position A1 should grow as the plasmid will be transferred from XL1-Blue to the Keio mutant, creating the donor strain (Kan^r^ F′ Tet^r^). The following day, the transconjugant (position A1) was pinned to a fresh LB TET KAN plate and allowed to grow overnight at 37°C. The plates were inspected for any strains that failed to grow, and 25 strains were manually mated (see Text S1). The purified transconjugants were pinned back to 96-well plates containing 125 μl of LB TET KAN and incubated at 37°C for approximately 20 h. Glycerol was added to the plates to a final concentration of 15% in a total volume of 175 μl. The Keio donor plates were frozen at −80°C.

### Donor Keio library screen.

The Keio donor plates were thawed at room temperature, and the HDA RoToR robot (Singer Ltd, United Kingdom) was used to transfer cells to an LB KAN TET preculture plate in duplicate, four replicates per plate (total, *n *= 8). A preculture plate of the recipient HA4 was made in the same way. After 16 h at 30°C, the two plates were pinned together onto LB TET CHL plates, creating 4 replicate matings per plate, and all appropriate negative controls were included (donors and recipients alone). A control mating of HA4 × HA14 was included in every fourth position to control for any spatial variation arising from plate position. Figure S6C details the pinning scheme. After pinning the two precultures together onto the selective mating plate (LB TET CHL), the pinned plate was immediately fixed in the scanner and the experiment was initiated. The secondary screen of 94 selected strains was performed in the same way, with 6 replicates distributed across each of three plates (*n *= 18). One hundred eight strains were not included in the primary screen but were done in high replication (*n* = 12) along with the secondary screen.

### Automated extraction of lag times and growth rates.

High-resolution population size growth curves were obtained using Epson Perfection V800 photo scanners (Epson Corporation, United Kingdom) and the Scan-o-Matic framework version 2.0 (14). Scanners were maintained in a single thermostatic (30°C), high-humidity cabinet to minimize light influx and evaporation. Experiments were run for 24 h, with automated transmissive scanning and signal calibration in 10-min intervals. Calibrated pixel intensities were transformed into population size measures by referencing to cell counts obtained by optical density measurements, using the conversion *y *= 2.128 × 10^−2^ *x*^5^ + 1.023 *x*^4^ + 11.47 *x*^3^ + 25.62 *x*^2^. Population growth curves were smoothed to remove noise using a Lowess-like weighted polynomial function (49). Poor-quality curves (0.25%), most commonly due to failed cell deposition (mispinning), were rejected following manual inspection. We segmented smoothed, log_2_ scale growth curves to identify an initial flat phase as a sequence of at least 3 data points with the required properties −0.02 < *d* < 0.02, where *d* is the first derivative. We next segmented the remaining part of the growth curves to identify the linear phase that corresponds to the largest increase in population size and extracted this value as growth rate. We extracted the lag time as the intercept between the initial flat and the linear phase, if the start of the linear phase occurs after the end of the initial flat phase. Details can be found in the work of Zackrisson (49); the code is available at https://github.com/Scan-o-Matic/scanomatic/blob/1b803ab5463f027cfe106034fffc60b5b5d3a9ff/scanomatic/data_processing/phases/features.py#L417-L457.

### Confirmation of mutant alleles by PCR.

Donor strains carrying the mutant Keio alleles were analyzed by standard colony PCR using the primers in Table S2 and the kanamycin cassette internal primer k1, which gives two bands if the gene has been replaced by the cassette and a single band if not (48). Control reactions were done on BW25113.

### Transductions.

P1 transduction of the Keio mutations to BW25113 was done as previously described (46) using kanamycin as the selective agent.

### Qβ phage sensitivity.

Strains to be tested were grown overnight in LB medium containing 10 mM MgCl_2_, 5 mM CaCl_2_, and appropriate antibiotics. One hundred fifty microliters of the overnight culture was added to 1 ml of fresh LB medium containing 10 mM MgCl_2_ and 5 mM CaCl_2_. Three milliliters of LB-soft agar (3 g/liter agar) was added, and the mixture was immediately poured onto an LB plate. A stock of phage Qβ (∼10^11^-PFU/ml stock concentration) was serially diluted in 10-fold steps from 10^−1^ to 10^−9^, and 10 μl of each dilution was spotted onto the bacterial lawn. Plates were incubated upside down overnight at 37°C, and plaques were counted the next day.

### Liquid mating assay.

Liquid mating assays were modified from the work of Anthony et al. (50). Cultures of each candidate, HA14 and HA4, were grown overnight in LB with appropriate antibiotics. The following day, the antibiotics were washed off and the cells were resuspended in 1 ml LB, prewarmed to 37°C. The washed cells were diluted 1:50 in prewarmed LB and grown to log phase. Recipient cells were adjusted to an optical density at 600 nm (OD_600_) of 3.0. Five hundred microliters of each candidate (or HA14 control) was mixed with 500 μl of HA4 and allowed to conjugate without shaking for 30 min at 37°C. After the incubation, conjugation was stopped by placing the cells on ice for 1 min followed by vigorous vortexing for 1 min, and thereafter, cells were kept on ice. Serial dilutions (diluted in 10-fold steps down to 10^−7^) of each conjugation mixtures were prepared in 1× M9 salts (6 g/liter Na_2_HPO_4_, 3 g/liter KH_2_PO_4_, 1 g/liter NH_4_Cl, and 0.5 g/liter NaCl). Ten microliters of each dilution was spotted twice on LB TET KAN plates and LB TET CHL and incubated at 37°C overnight to quantitate the number of donors and transconjugants, respectively. Conjugation frequency was calculated as the number of transconjugants per donor. The calculated frequency was then normalized to the mean conjugation frequency of the control matings (HA4 × HA14) on the same day and expressed as a ratio of the control. The average conjugation frequency for the control was 0.35 (35%) ± 0.22 transconjugants/donor. Raw data are available at https://github.com/annefarewell/Conjugation-factors-F-plasmid.

### Growth rate measurements.

The growth rate of the Keio donor strains used in the secondary screen was measured. They were pinned, in 12 biological replicates, from frozen 96-well culture stocks onto a preculture plate containing LB KAN TET, grown overnight, and then pinned onto the same medium. These plates were scanned and growth rates were extracted as described above.

### UV sensitivity.

Strains were grown overnight in LB with appropriate antibiotic selection and then diluted 20 times. They were then grown at 37°C until they reached an OD_600_ of around 0.5. A 10-fold dilution series was done on each strain to 10^−6^ in M9 minimal medium lacking glucose. Ten microliters of each dilution for each strain was then spotted on 6 different LB plates. These plates were then exposed to UV light at a standard distance for 0 s, 5 s, 10 s, 15 s, 20 s, and 25 s, respectively. After exposure, the plates were immediately covered with aluminum foil to stop the E. coli from using natural light to repair the UV damage through photoreactivation. These plates were then placed at 37°C overnight, and colonies were counted the following day. The control strain (wild type) showed 90% survival at 15 s, compared to the *uvrD* mutant that had <0.00001% survival.

### Statistics.

Statistical analysis was performed in R. Tests used are indicated in the text or figure legends.

### Data availability.

Scan-o-Matic software and scripts are available at https://github.com/Scan-o-Matic/. Extracted data from the screens and other raw data are available at https://github.com/annefarewell/Conjugation-factors-F-plasmid.
