# Supplementary figures and images for: A High-Throughput Method for Screening for Genes Controlling Bacterial Conjugation of Antibiotic Resistance
Source: mSystems. 2020 Dec 22;5(6):e01226-20. doi: 10.1128/mSystems.01226-20 (PMC7762799; doi:10.1128/mSystems.01226-20)

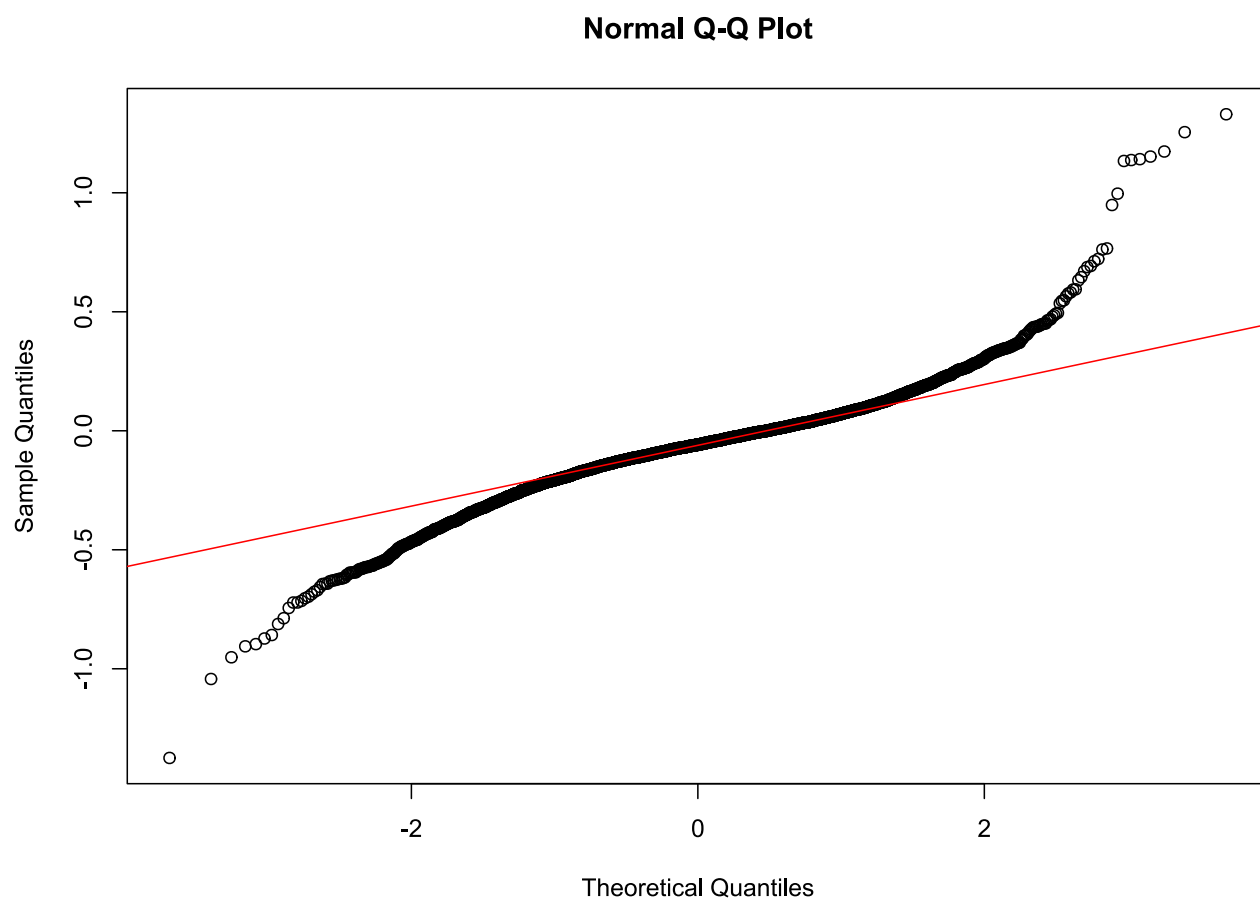

Figure S1

Supplement: FIG S1 [file mSystems.01226-20-sf001.pdf]

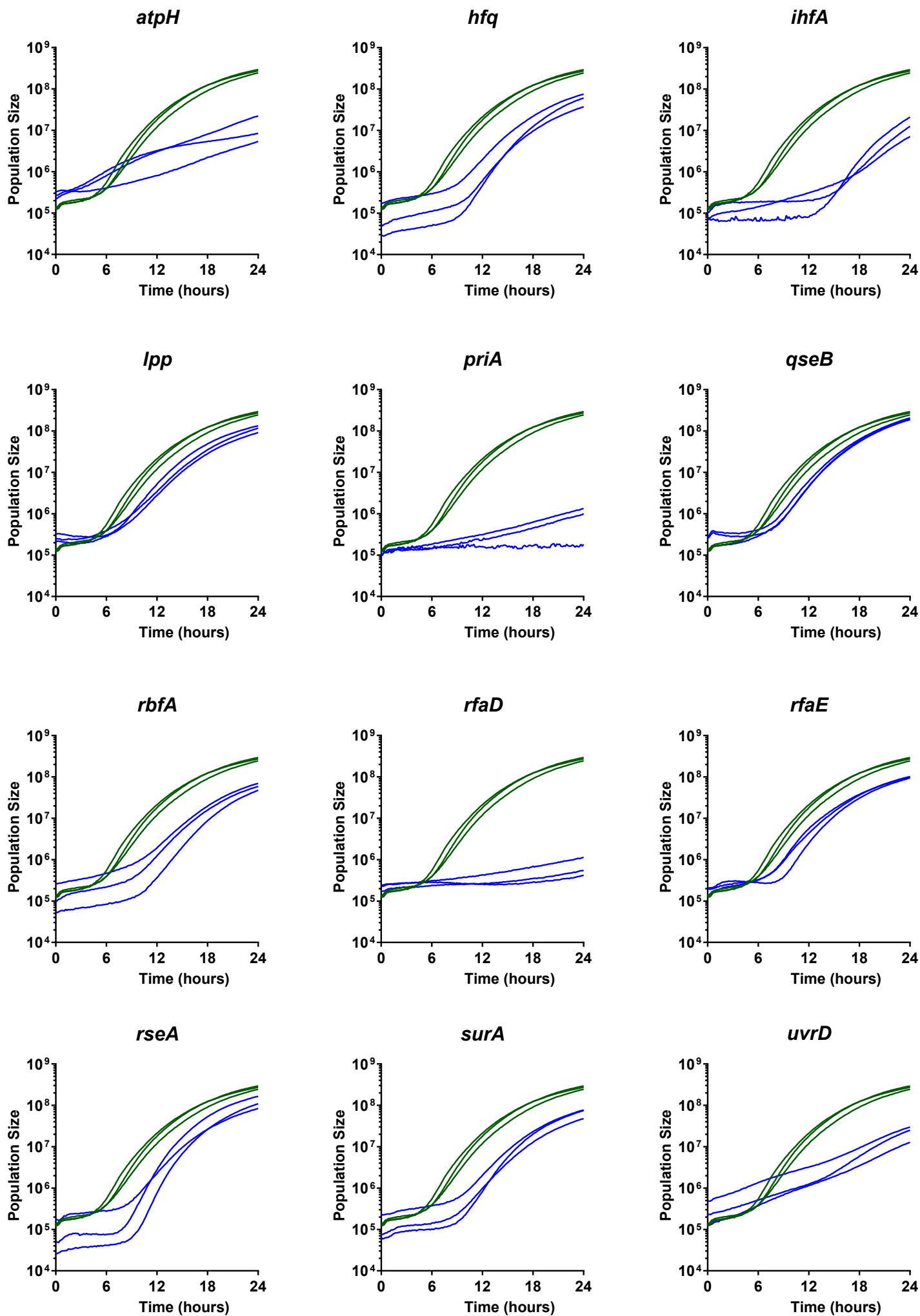

Figure S2

Supplement: FIG S2 [file mSystems.01226-20-sf002.pdf]

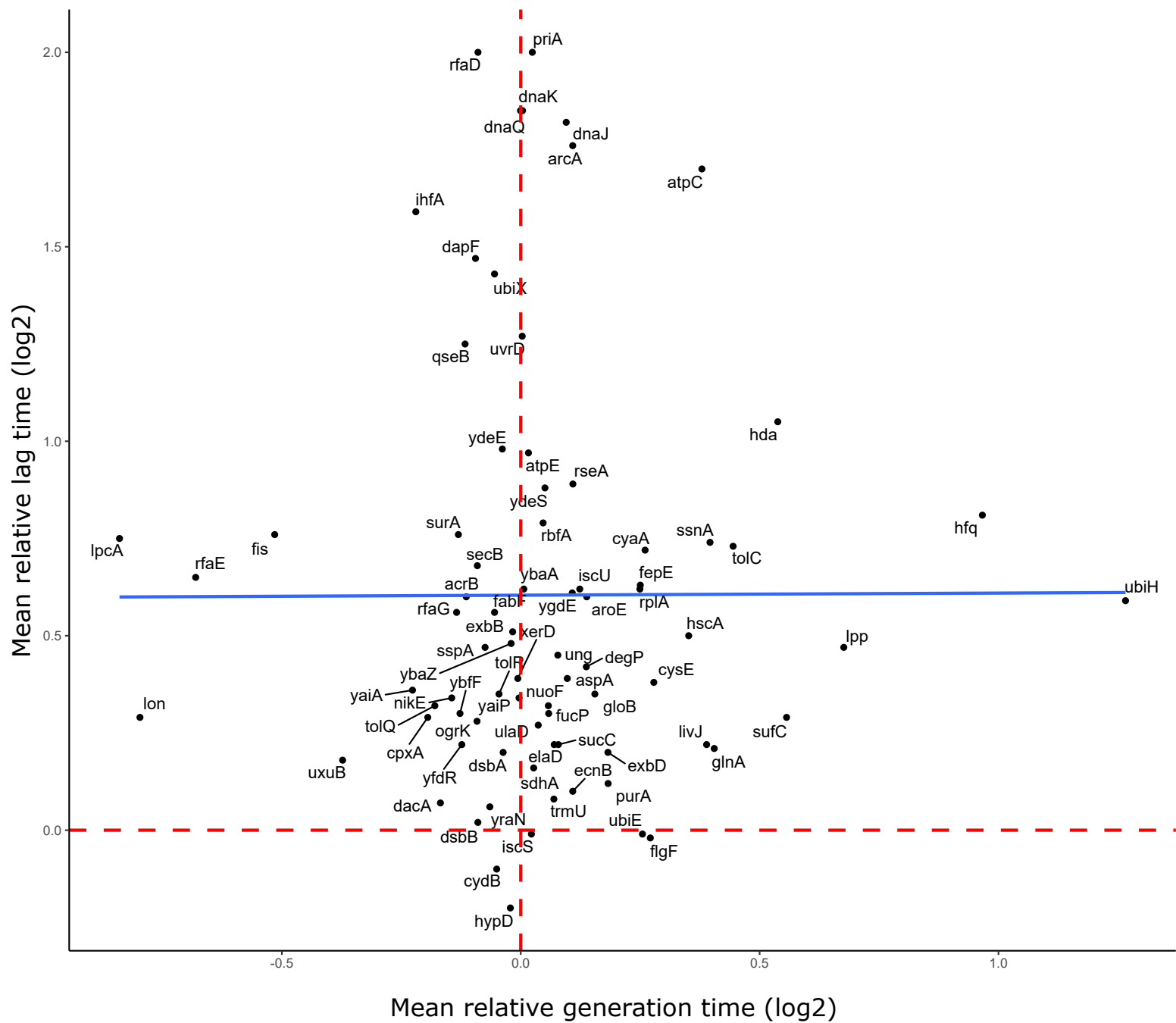

Supplement: FIG S3 [file mSystems.01226-20-sf003.pdf]

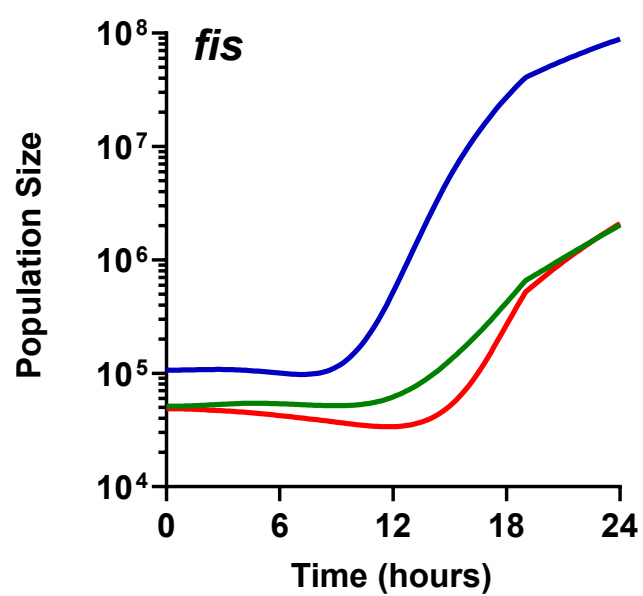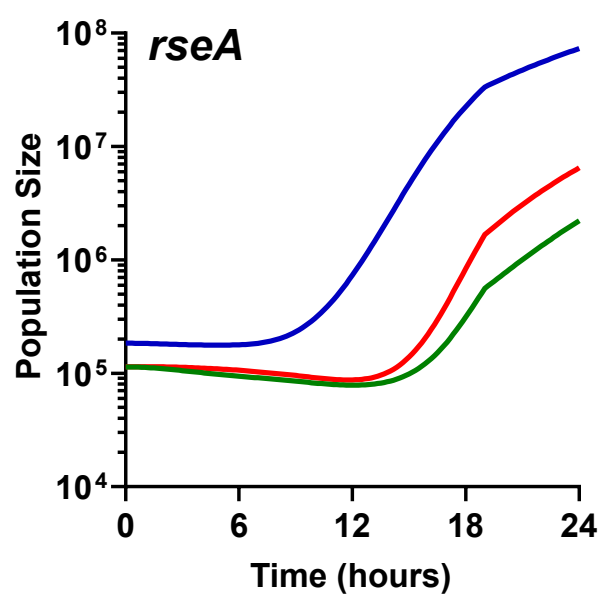

Figure S4

Supplement: FIG S4 [file mSystems.01226-20-sf004.pdf]

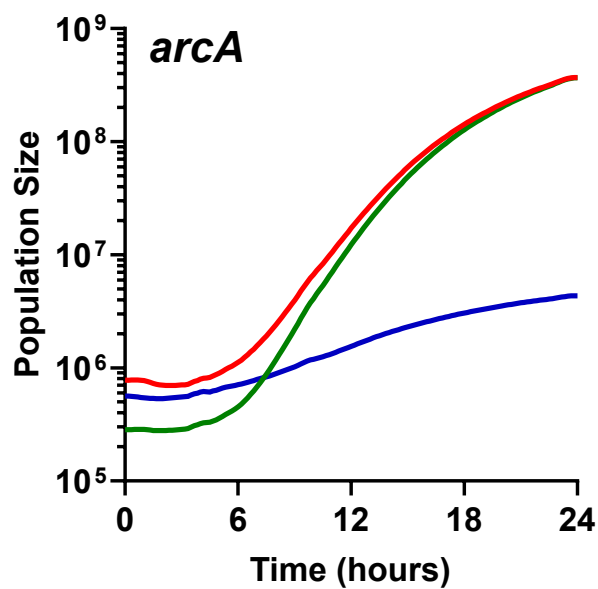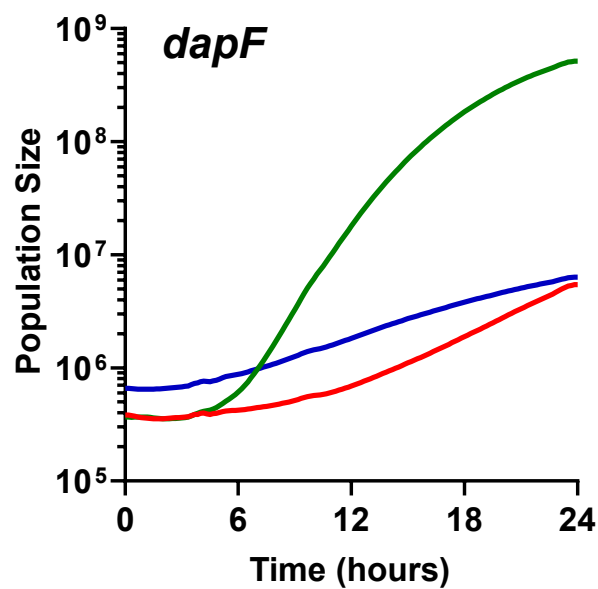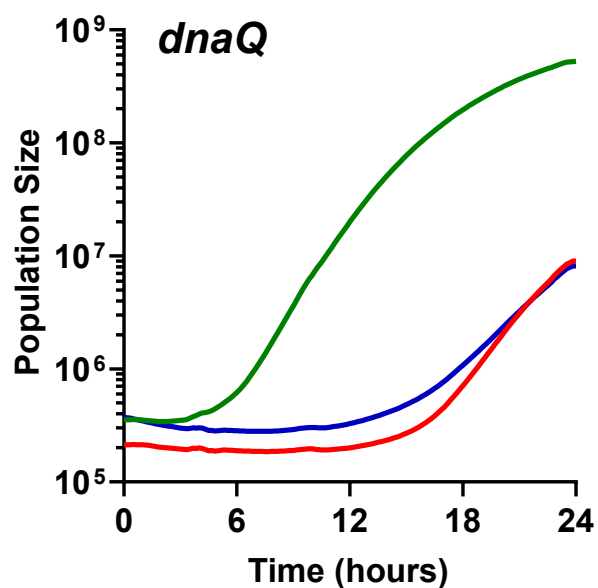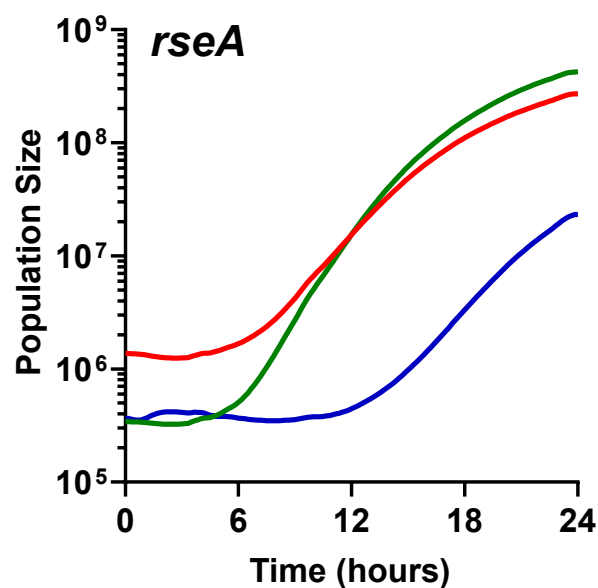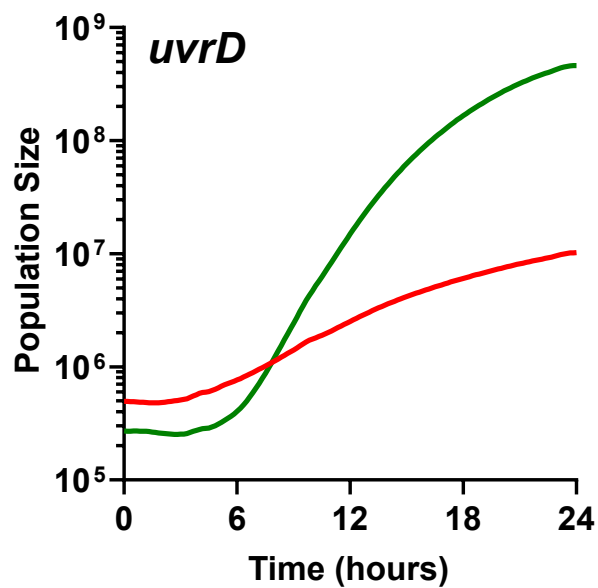

Figure S5

Supplement: FIG S5 [file mSystems.01226-20-sf005.pdf]

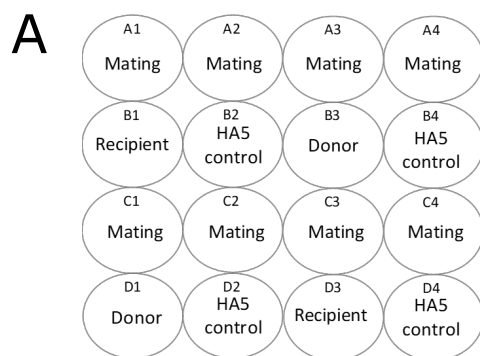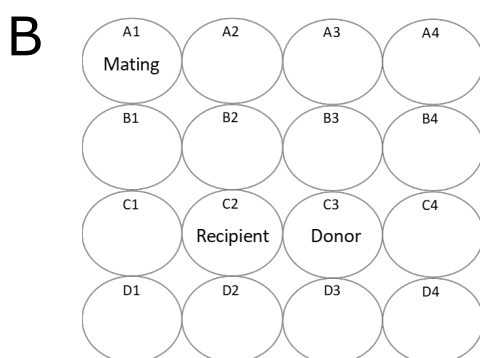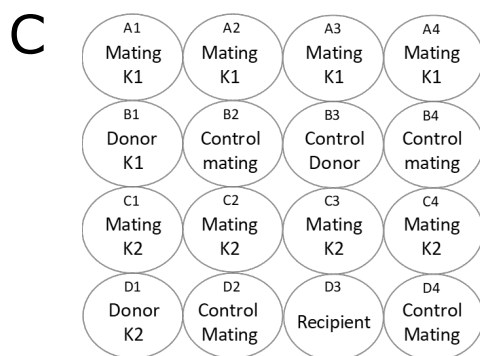

Figure S6

Supplement: FIG S6 [file mSystems.01226-20-sf006.pdf]
