# Supplementary material for: A High-Throughput Method for Screening for Genes Controlling Bacterial Conjugation of Antibiotic Resistance
Source: mSystems. 2020 Dec 22;5(6):e01226-20. doi: 10.1128/mSystems.01226-20 (PMC7762799; doi:10.1128/mSystems.01226-20)
Supplement: TABLE S1 [file mSystems.01226-20-st001.docx]

|  |  |  | **Initial Screen** | |  | **Secondary Screen^c^** | | |  |  |
| --- | --- | --- | --- | --- | --- | --- | --- | --- | --- | --- |
| **Rank^a^** | **Gene** | **Gene Product^b^** | **median** | **mean** | **SE.mean** | **median** | **mean** | **SE.mean** | | **Significance^d^** |
| 1* | *uvrD* | ssDNA translocase and dsDNA helicase - DNA helicase II | - | - | - | 2.00 | 1.27 | 0.24 | | 1.45E-03 |
| 1* | *rfaD* | ADP-L-glycero-D-mannoheptose 6-epimerase | - | - | - | 2.00 | 2.00 | 0.00 | | 1.03E-04 |
| 1* | *arcA* | DNA-binding transcriptional dual regulator ArcA | - | - | - | 2.00 | 1.76 | 0.13 | | 2.35E-04 |
| 1* | *dnaQ* | DNA polymerase III subunit ε | - | - | - | 2.00 | 1.85 | 0.04 | | 3.08E-04 |
| 1* | *atpH* | ATP synthase F1 complex subunit δ | - | - | - | 2.00 | 1.95 | 0.05 | | 1.41E-04 |
| 1* | *priA* | primosome factor N' | - | - | - | 2.00 | 2.00 | 0.00 | | 1.03E-04 |
| - | *crp* | DNA-binding transcriptional dual regulator CRP | N.D. | N.D. | N.D. | 2.00 | 1.49 | 0.22 | | 2.94E-03 |
| 8* | *ihfA* | integration host factor subunit α | 1.33 | 1.33 | - | 2.00 | 1.59 | 0.12 | | 4.30E-04 |
| 11* | *surA* | peptidyl-prolyl cis-trans isomerase SurA | 1.17 | 1.17 | - | 0.75 | 0.76 | 0.03 | | 4.24E-05 |
| 12 | *dnaK* | chaperone protein DnaK | 1.14 | 1.15 | 0.02 | 2.00 | 1.85 | 0.06 | | 3.08E-04 |
| 13* | *dapF* | diaminopimelate epimerase | 1.13 | 1.13 | - | 2.00 | 1.47 | 0.21 | | 4.76E-04 |
| 14 | *dsbA* | protein disulfide oxidoreductase - DsbA | 0.99 | 1.00 | 0.08 | 0.17 | 0.20 | 0.19 | | N.S. |
| 15* | *rfaH* | transcription antiterminator RfaH | 0.95 | 0.95 | 0.11 | 2.00 | 1.83 | 0.12 | | 2.08E-03 |
| 16 | *fis* | DNA-binding transcriptional dual regulator Fis | 0.89 | 0.76 | 0.10 | 0.82 | 0.76 | 0.05 | | 4.24E-05 |
| 17 | *iscU* | scaffold protein for iron-sulfur cluster assembly | 0.76 | 0.72 | 0.05 | 0.59 | 0.62 | 0.04 | | 4.24E-05 |
| 18 | *dnaJ* | chaperone protein DnaJ | 0.73 | 0.55 | 0.31 | 2.00 | 1.82 | 0.14 | | 2.24E-04 |
| 19 | *ssnA* | putative aminohydrolase | 0.72 | 0.71 | 0.05 | 0.63 | 0.74 | 0.09 | | 4.24E-05 |
| 20 | *hda* | inibitor of reinitiation of DNA replication | 0.72 | 0.69 | 0.02 | 0.93 | 1.05 | 0.09 | | 4.65E-04 |
| 23 | *iscS* | cysteine desulfurase | 0.65 | 0.65 | 0.04 | -0.08 | -0.01 | 0.14 | | N.S. |
| 24 | *atpC* | ATP synthase F1 complex subunit ε | 0.63 | 0.59 | 0.05 | 2.00 | 1.70 | 0.17 | | 3.08E-04 |
| 25 | *hscA* | iron-sulfur cluster biosynthesis chaperone HscA | 0.63 | 0.63 | 0.04 | 0.48 | 0.50 | 0.04 | | 4.24E-05 |
| 26 | *rseA* | anti-sigma factor | 0.61 | 0.59 | 0.03 | 0.86 | 0.89 | 0.04 | | 4.24E-05 |
| 27 | *tolR* | Tol-Pal system protein TolR | 0.60 | 0.58 | 0.03 | 0.22 | 0.35 | 0.15 | | 5.86E-03 |
| 28 | *hfq* | RNA-binding protein Hfq | 0.58 | 0.56 | 0.01 | 0.76 | 0.81 | 0.08 | | 4.24E-05 |
| 29 | *ydeE* | dipeptide exporter | 0.57 | 0.49 | 0.07 | 0.41 | 0.98 | 0.22 | | 1.26E-03 |
| 31 | *cysE* | serine acetyltransferase | 0.52 | 0.54 | 0.04 | 0.36 | 0.38 | 0.02 | | 4.24E-05 |
| 33 | *sufC* | Fe-S cluster scaffold complex subunit SufC | 0.49 | 0.45 | 0.04 | 0.17 | 0.29 | 0.11 | | 1.91E-04 |
| 34 | *secB* | SecB chaperone | 0.48 | 0.46 | 0.03 | 0.45 | 0.68 | 0.21 | | 6.50E-03 |
| 35 | *cpxA* | sensory histidine kinase CpxA | 0.48 | 0.49 | 0.04 | 0.22 | 0.29 | 0.11 | | 5.86E-03 |
| - | *aroD* | 3-dehydroquinate dehydratase | N.D. | N.D. | N.D. | 0.47 | 0.46 | 0.04 | | 9.21E-04 |
| 37 | *fabF* | β-ketoacyl-[acyl carrier protein] synthase II | 0.45 | 0.44 | 0.04 | 0.35 | 0.56 | 0.15 | | 6.54E-04 |
| 38 | *cydB* | cytochrome bd-I ubiquinol oxidase subunit II | 0.45 | 0.43 | 0.12 | -0.37 | -0.10 | 0.18 | | N.S. |
| 39 | *uxuB* | D-mannonate oxidoreductase | 0.45 | 0.33 | 0.10 | 0.17 | 0.18 | 0.15 | | 2.07E-02 |
| 40 | *lpp* | murein lipoprotein | 0.45 | 0.45 | 0.01 | 0.46 | 0.47 | 0.02 | | 4.24E-05 |
| 41 | *rplA* | 50S ribosomal subunit protein L1 | 0.44 | 0.47 | 0.04 | 0.59 | 0.62 | 0.04 | | 4.24E-05 |
| 42 | *fepE* | polysaccharide co-polymerase family protein FepE | 0.44 | 0.45 | 0.05 | 0.44 | 0.63 | 0.12 | | 4.65E-04 |
| 43 | *dacA* | D-alanyl-D-alanine carboxypeptidase DacA | 0.44 | 0.41 | 0.03 | 0.22 | 0.07 | 0.09 | | N.S. |
| 44 | *gloB* | hydroxyacylglutathione hydrolase GloB | 0.44 | 0.44 | 0.05 | 0.45 | 0.35 | 0.07 | | 3.02E-03 |
| 45 | *trmU* | tRNA-specific 2-thiouridylase (mnmA) | 0.43 | 0.42 | 0.03 | 0.20 | 0.08 | 0.10 | | N.S. |
| 46 | *rfaG* | lipopolysaccharide glucosyltransferase I (WaaG) | 0.43 | 0.38 | 0.06 | 0.56 | 0.56 | 0.11 | | 2.46E-04 |
| 47 | *yraN* | UPF0102 family protein YraN | 0.43 | 0.44 | 0.03 | 0.11 | 0.06 | 0.07 | | N.S. |
| 48 | *ubiH* | 2-octaprenyl-6-methoxyphenol hydroxylase | 0.42 | 0.37 | 0.06 | 0.32 | 0.59 | 0.16 | | 4.65E-04 |
| 49 | *qseB* | DNA-binding transcriptional activator QseB | 0.42 | 0.37 | 0.05 | 1.41 | 1.25 | 0.19 | | 4.65E-04 |
| 50 | *dsbB* | protein disulfide oxidoreductase - DsbB | 0.42 | 0.24 | 0.13 | 0.05 | 0.02 | 0.04 | | N.S. |
| 51 | *hypD* | scaffold protein for assembly of the Fe-(CN)2CO cofactor | 0.42 | 0.47 | 0.03 | -0.14 | -0.20 | 0.08 | | 1.67E-02 |
| 53 | *lpcA* | D-sedoheptulose 7-phosphate isomerase | 0.40 | 0.16 | 0.22 | 0.51 | 0.75 | 0.12 | | 4.65E-04 |
| 55 | *livJ* | branched chain aa/phe ABC transporter periplasmic binding protein | 0.39 | 0.43 | 0.03 | 0.23 | 0.22 | 0.03 | | 1.03E-04 |
| 56 | *ygdE* | 23S rRNA 2'-O-ribose C2498 methyltransferase | 0.39 | 0.40 | 0.01 | 0.36 | 0.61 | 0.16 | | 7.06E-04 |
| 57 | *rbfA* | 30S ribosome binding factor | 0.39 | 0.40 | 0.03 | 0.79 | 0.79 | 0.05 | | 4.24E-05 |
| 58 | *acrB* | multidrug efflux pump RND permease AcrB | 0.39 | 0.36 | 0.02 | 0.50 | 0.60 | 0.09 | | 4.24E-05 |
| 59 | *ybaZ (atl)* | DNA base-flipping protein | 0.38 | 0.41 | 0.04 | 0.40 | 0.48 | 0.10 | | 7.27E-05 |
| 60 | *ung* | uracil-DNA glycosylase | 0.38 | 0.38 | 0.03 | 0.46 | 0.45 | 0.03 | | 4.24E-05 |
| 62 | *sdhA* | succinate:quinone oxidoreductase, FAD binding protein | 0.38 | 0.40 | 0.04 | 0.21 | 0.16 | 0.09 | | N.S. |
| 63 | *tolC* | outer membrane channel TolC | 0.38 | 0.37 | 0.03 | 0.60 | 0.73 | 0.09 | | 4.24E-05 |
| 64 | *aspA* | aspartate ammonia-lyase | 0.37 | 0.39 | 0.04 | 0.20 | 0.39 | 0.19 | | N.S. |
| 67 | *exbB* | Ton complex subunit ExbB | 0.36 | 0.33 | 0.04 | 0.36 | 0.51 | 0.14 | | 4.65E-04 |
| 68 | *degP* | periplasmic serine endoprotease DegP | 0.35 | 0.34 | 0.02 | 0.41 | 0.42 | 0.04 | | 7.27E-05 |
| 70* | *flgF* | flagellar basal-body rod protein FlgF | 0.35 | 0.37 | 0.06 | -0.12 | -0.02 | 0.20 | | N.S. |
| 71 | *yfdR* | CPS-53 (KpLE1) prophage; 5'-deoxynucleotidase | 0.35 | 0.34 | 0.03 | 0.20 | 0.22 | 0.04 | | 1.41E-04 |
| 72 | *ecnB* | bacteriolytic entericidin B lipoprotein | 0.35 | 0.35 | 0.02 | 0.09 | 0.10 | 0.06 | | N.S. |
| 73 | *yaiP* | putative glucosyltransferase | 0.34 | 0.35 | 0.03 | 0.33 | 0.34 | 0.03 | | 4.24E-05 |
| 74 | *rfaE* | fused heptose 7-phosphate kinase/heptose 1-phosphate adenyltransferase | 0.34 | 0.34 | 0.02 | 0.65 | 0.65 | 0.05 | | 4.24E-05 |
| 75 | *ubiE* | bifunctional 2-octaprenyl-6-methoxy-1,4-benzoquinone methylase and S-adenosylmethionine:2-DMK methyltransferase | 0.34 | 0.35 | 0.05 | 0.06 | -0.01 | 0.08 | | N.S. |
| 78 | *sspA* | stringent starvation protein A | 0.34 | 0.27 | 0.06 | 0.40 | 0.47 | 0.10 | | 7.27E-05 |
| 80 | *atpE* | ATP synthase Fo complex subunit c | 0.34 | 0.32 | 0.03 | 0.66 | 0.97 | 0.21 | | 1.49E-03 |
| 82 | *elaD* | protease ElaD | 0.33 | 0.33 | 0.01 | 0.27 | 0.22 | 0.06 | | 5.10E-03 |
| 83 | *nikE* | Ni(2+) ABC transporter ATP binding subunit NikE | 0.33 | 0.32 | 0.03 | 0.22 | 0.34 | 0.15 | | 5.86E-03 |
| 85 | *ybaA* | conserved protein YbaA | 0.33 | 0.35 | 0.02 | 0.36 | 0.62 | 0.19 | | 3.02E-03 |
| 86 | *ulaD* | 3-keto-L-gulonate-6-phosphate decarboxylase UlaD | 0.33 | 0.33 | 0.06 | 0.19 | 0.27 | 0.11 | | 1.22E-03 |
| 88 | *fucP* | L-fucose:H+ symporter | 0.33 | 0.33 | 0.02 | 0.22 | 0.30 | 0.11 | | 3.08E-04 |
| 90 | *cyaA* | adenylate cyclase | 0.33 | 0.35 | 0.09 | 0.20 | 0.72 | 0.28 | | N.S. |
| 93 | *tolQ* | Tol-Pal system protein TolQ | 0.32 | 0.36 | 0.07 | 0.40 | 0.32 | 0.13 | | 1.83E-02 |
| 94 | *exbD* | Ton complex subunit ExbD | 0.32 | 0.34 | 0.07 | 0.06 | 0.20 | 0.17 | | N.S. |
| 102 | *nuoF* | NADH:quinone oxidoreductase subunit F | 0.30 | 0.35 | 0.06 | 0.18 | 0.32 | 0.15 | | 1.76E-02 |
| 118 | *lon* | Lon protease | 0.27 | 0.29 | 0.07 | 0.22 | 0.29 | 0.11 | | 1.22E-03 |
| 130 | *ogrK* | prophage P2 late control protein OgrK | 0.26 | 0.32 | 0.06 | 0.19 | 0.28 | 0.11 | | 8.23E-04 |
| 138 | *xerD* | site-specific recombinase | 0.25 | 0.31 | 0.07 | 0.07 | 0.39 | 0.18 | | 1.76E-02 |
| 152 | *ubiX* | flavin prenyltransferase | 0.24 | 0.28 | 0.04 | 2.00 | 1.43 | 0.26 | | 2.45E-03 |
| 158 | *aroE* | shikimate dehydrogenase | 0.24 | 0.26 | 0.05 | 0.17 | 0.60 | 0.22 | | 7.34E-03 |
| 164 | *ybfF* | esterase | 0.24 | 0.25 | 0.04 | 0.29 | 0.30 | 0.03 | | 4.24E-05 |
| 196 | *yaiA* | protein YaiA | 0.21 | 0.20 | 0.02 | 0.28 | 0.36 | 0.11 | | 1.41E-04 |
| 202 | *glnA* | glutamine synthetase | 0.21 | 0.17 | 0.04 | 0.10 | 0.21 | 0.11 | | 1.83E-02 |
| 203 | *ydeS* | putative fimbrial protein YdeS | 0.21 | 0.23 | 0.02 | 0.52 | 0.88 | 0.21 | | 3.02E-03 |
| 230 | *purA* | adenylosuccinate synthetase | 0.19 | 0.20 | 0.05 | 0.02 | 0.12 | 0.12 | | N.S. |
| 236* | *sucC* | succinyl-CoA synthetase subunit β | 0.18 | 0.18 | 0.15 | -0.04 | 0.22 | 0.21 | | N.S. |
