# Supplementary material for: A High-Throughput Method for Screening for Genes Controlling Bacterial Conjugation of Antibiotic Resistance
Source: mSystems. 2020 Dec 22;5(6):e01226-20. doi: 10.1128/mSystems.01226-20 (PMC7762799; doi:10.1128/mSystems.01226-20)
Supplement: TABLE S2 [file mSystems.01226-20-st002.docx]

| **Deleted genes**  **checked by PCR** | **Primer forward (5' -> 3')** | **Primer reverse (5' -> 3')** |
| --- | --- | --- |
| *uvrD* | TTTCCCGGTTGGCATCTCTG | ATCCGGCCTACATGACGTTG |
| *rfaD* | TGCAATTAGCATCCTTGCACC | AATAATGGACGGCACCATGC |
| *arcA* | ACACTGTCGGGTCCTGAGGGA | ACCAGTGTGCTGGTGGTGGC |
| *dnaQ* | ATTGCCCAGACACGAACCAT | TCGACCTTCGTCAACGGTTT |
| *dapF* | TCCACGGTGCCGGATAAAAA | CGCGCGCATTACGGATAAAA |
| *ihfA* | ATCCTGCAAGATACCAGCCG | GCTGAAGTGTCATGGCGTTG |
| *dsbA* | GGGAAGATTACTGGCTGCGA | AGCGGCAGGATGCATTATCA |
| *dnaJ* | ATGACGATGTTGTCGACGCT | CTTATGGGAGTGATCCCCGC |
| *ssnA* | TAACAACGTACCACCGGAGC | GACAGAGTCGGGCCTGAAAA |
| *rseA* | CACGTATCTTCCGAGCGAGG | CCGAGGCGTTAGCAGAGAAT |
| *iscS* | TCGACGTTAAGTTACGCGCT | CGACGTTCTCGTCGTTGTTG |
| *tolR* | GTTATGGCCTACAACCGCCT | TGTCGTTTTGTTCGGTTGCC |
| *hfq* | TGCTATCGCAGGCTGAATGT | GCCTGCTCACCAGCATCATA |
| *secB* | ATTGACGCACAGCACATTGG | ACAACCTCGTGGCCATTTCT |
| *sufC* | CCGTTGGAATTTGCCGTTGA | TAAATGCTGCTGTGCTTGCG |
| *cpxA* | CAGGAAGTGTTGGGCAAACG | TGTGGGGAAAATAACCCCCG |
| *fabF* | AAATCACCACCGTTCAGGCT | AGTGTGGCAGCATGTTCACT |
| *fepE* | TGTTGCATTCAGTGGAAGGA | GAGCGCATTTATGGTCTGCG |
| *dacA* | AGCGTAAAGCAGGCATCTGA | ATAGCAACTCCCGCCAGAAG |
| *gloB* | AGGAGCCACGACAGTTTGAG | AGACGACCGATCATAACGGC |
| *trmU* | ATCCGCTGGAGATGATTGGC | GCGTGCCGACTGACAAATAC |
| *rfaG* | CTGGGCGGGGAATTATCAGAA | ACGACGAGTCTCCAGTTCAC |
| *qseB* | CAACACGGTTTACTGGCAGC | CAGGCGACAAAGCTGGAAAG |
| *dsbB* | AATGCCCGGTTTGCCTTTTC | CAATGGCAGATGAAGCGAGC |
| *lpcA* | TACTTCTCGCTTTTGGCGGT | GCATGATAAGACGCGTCAGC |
| *livJ* | ATGCTGCTAAAGCACGGGT | AGGCCTACAATGTGTGTTGC |
| *sdhA* | GCTGCAACTGGTGATTGTCG | TACGCGGAGCATCATCAACA |
| *tolC* | ACGTAACGCCAACCTTTTGC | TTTGTCTTCCGGGACCAGTG |
| *flgF* | CAGATCCTCAACACGCTGGT | CGATAAACTTGCGCATCGGG |
| *rfaE* | TGTCGGAGGATTGCTTCACC | TCCTCCCTTACCCTTGTTCCT |
| *sspA* | GCCCAAATGTCGGGTATTGC | CGTCACATCCACCACCAGG |
| *fucP* | TATGAAGCCCGAACTCGCTG | ACCGGGCGGATACCAATTTT |
| *tolQ* | CTGGTTGTTTGCGTTGACCC | ACGTCCAGCAACGGTACAAT |
| *nuoF* | ATTTGATGGCCGCTTTACGC | CGCTCCGTTGACCTCGTATT |
| *glnA* | GTGCGCATGATAACGCCTTT | CCGTGTAGGCCGGATAAGAC |
| *sucC* | CAAGCGATGCCTGATGTGAC | GTTACGCCGCCAACCATTTT |
