# Supplementary material for: A High-Throughput Method for Screening for Genes Controlling Bacterial Conjugation of Antibiotic Resistance
Source: mSystems. 2020 Dec 22;5(6):e01226-20. doi: 10.1128/mSystems.01226-20 (PMC7762799; doi:10.1128/mSystems.01226-20)
Supplement: TABLE S3 [file mSystems.01226-20-st003.docx]

| **Strain** | **EOP** |
| --- | --- |
| BW25113/F’ (positive control) | +++ |
| Δ*argC*/F’ (positive control) | +++ |
| Δ*arcA*/F’ | 0 |
| Δ*dapF*/F’ | 0 |
| Δ*lpp*/F’ | +++ |
| Δ*fabF*/F’ | +++ |
| Δ*qseB*/F’ | 0 |
| Δ*dsbA*/F’ | +++ |
| Δ*surA*/F’ | +++ |
| Δ*rseA*/F’ | +++ |
| Δ*tolR*/F’ | +++ |
| Δ*fis*/F’ | +++ |
| Δ*uvrD*/F’ | +++ |
| Δ*dnaQ*/F’ | 0 |
| BW25113 (negative control) | 0 |

Table S3.
