# Supplementary material for: A High-Throughput Method for Screening for Genes Controlling Bacterial Conjugation of Antibiotic Resistance
Source: mSystems. 2020 Dec 22;5(6):e01226-20. doi: 10.1128/mSystems.01226-20 (PMC7762799; doi:10.1128/mSystems.01226-20)
Supplement: TEXT S1 [file mSystems.01226-20-s0001.docx]

**Manual construction of selected donor Keio strains**

During robotic construction of the Keio donor library, we noted 26 strains that did not produce Kan^R^ Tet^R^ transconjugants. One of these Keio strains (*thyA*) was not viable. The remainder (see below) were patched together on an LB plate together with XL1-Blue and grown for 16 hours at 37^o^C to allow mating. We then streaked for single colonies from the patches onto LB KAN TET to select for transconjugants. In this way we were able to obtain transconjugants of all of these strains. We note that it is possible these strains are deficient in acting as recipients but have not characterized them further.

**Keio strains subjected to manual mating.**

| *acs* | *dnaQ* | *recB* | *tesB* | *yajB* |
| --- | --- | --- | --- | --- |
| *aes* | *fabH* | *rfaC* | *ubiG* | *yfgA* |
| *atoB* | *idnK* | *rfaD* | *uxuA* | *yghG* |
| *atpE* | *lpcA* | *rfaF* | *wzxC* | *yghR* |
| *dnaK* | *priA* | *sfsA* | *yaiE* | *ylbA* |

**Construction of complementation plasmids**

Primers were designed for each gene based on information in Ecocyc^1^ with restriction sites for HindIII or XhoI added (see Table below) and ordered from Thermo Scientific Inc. The pBBR1 replicon-Gen^R^ cassette was amplified from pTA-mob^2^ to form the backbone of the plasmid with matching restriction enzyme sites. PCR was done with enzyme Q5 according to the manufacturers’ instructions (NEB, Inc). Digestion was followed by gel purification and ligation using T4 DNA ligase according to the manufacturer’s instructions. Ligations were first transformed into NEB10β (NEB, Inc.) cells and selected on 20 μg/ml gentamycin. Plasmids were then extracted and transformed into the appropriate Keio strain using standard methods^3^. As a control the vector backbone was also ligated and transformed into the same strains. Inserts were sequenced at Eurofins, Inc.

**PCR primers used in cloning.**

| **Primer** | **Sequence** |
| --- | --- |
| XhoI pTA-mob backbone | ATAGCTCGAGATTAATCCACCTGGCGGCG |
| HindIII pTA-mob backbone | GAGCAAGCTTCCTAGGTTAAACGCCTGGTGC |
| HindIII *dapF* forward | GAGCAAGCTTTGCGATGATAGAAAGCAGAAAGC |
| XhoI *dapF* reverse | ATAGCTCGAGAGATAATCGACAACCGCCCG |
| HindIII *uvrD* forward | GAGCAAGCTTACCGGAAAATGGCGATCTGA |
| XhoI *uvrD* reverse | ATAGCTCGAGCGTGGCAACGCTATCCTTTT |
| HindIII *dnaQ* forward | GAGCAAGCTTATACGGTTGTTGGTGGTGCG |
| XhoI *dnaQ* reverse | ATAGCTCGAGTCGTCAACGGTTTTTCTCATCT |
| HindIII *rseA* forward | GAGCAAGCTTGTAAATACAGCGAAAAATTACCTGG |
| XhoI *rseA* reverse | ATAGCTCGAGGCGGGAGTGGCCGAG |
| HindIII *arcA* forward | GAGCAAGCTTCGACAATCCGCAGATCGCTA |
| XhoI *arcA* reverse | ATAGCTCGAGTCGACTGGTATGCGTTTTCAC |

**Pinning and conjugation of complemented strains**

Strains carrying the appropriate plasmids were pinned and mated to strain HA4 as described in the main text except that precultures for the donor strains contained gentamycin in addition to kanamycin and tetracycline.

**References**

1. Keseler, I. M. *et al.* The EcoCyc database: reflecting new knowledge about Escherichia coli K-12. *Nucleic Acids Res* **45**, D543–D550 (2017).

2. Strand, T. A., Lale, R., Degnes, K. F., Lando, M. & Valla, S. A New and Improved Host-Independent Plasmid System for RK2-Based Conjugal Transfer. *PLOS ONE* **9**, e90372 (2014).

3. Sambrook, J. & Russell, D. W. *Molecular Cloning: A Laboratory Manual*. (CSHL Press, 2001).
